# Emphasizing multiprofessional collaboration to meet the icu patients nutrition needs

**DOI:** 10.1186/2197-425X-3-S1-A586

**Published:** 2015-10-01

**Authors:** O Reiersdal, AH Pripp, G Rohde

**Affiliations:** University of Agder, Health and Sport Science, Kristiansand, Norway; Sorlandet Hospital HF, Kristiansand, Norway

## Introduction

Many studies have shown that the standard of the nutrition of ICU-patients influence the patient results, measured by ICU-LOS (length of stay), hospital-LOS and mortality. The energy deficit accumulating during the first days of the ICU stay may play an important role for the patient results. After implementation of a multi-professional ICU nutrition protocol, including a broadly teaching/information phase for both nurses and anesthesiologists, we conducted a retrospective study.

## Aim

The aim of the study was to evaluate the implementation of the ICU nutrition guidelines measured by prescribed and given kcal/kg/day, and to investigate the physicians’ and nurses’ compliance to the new guideline. The intervention: The nurses should drive the nutrition protocol, which means that they should assess the infusion rates, to reach the target of the kcal prescription.

## Methods

We conducted a retrospective study, with pre and post measures due to the implementation. Sample: Randomly selected patients in a 5-bed ICU with mixed surgical and medical patients at a medium-sized hospital in Norway. Inclusion criteria: Random adults ICU patients (over 18 years) on invasive ventilation support for minimum two days. We ended up with 665 days, which were the days of 45 patients. 364 days (of 25 patients) before the implementation and 301 days (of 20 patients) after the implementation. The average age in both groups were 65 years, and 49% woman and 51% men. The average ICU stay was 8 days, ranging from 2 to 25 days.

## Results

There was no significant difference from day 7-25, but from day 1-5 there were significant better results in nutrition of the patients measured in minimum kcal/day that was set to 25 kcal/kg/day (p < 0.001). Mean difference was 4.5 kcal/kg/day. The patients in the intervention group received mean 25/kg/day and the number for the control group was 20 kcal/kg/day. No significant differences in LOS, ventilator days or mortality.

We found no significant findings between the intervention group and the control group, when it came to deficit between what prescribed by the physicians and what was actually given by the nurses. After the implementation all the nurses were supposed to register and document the deficit every morning, but that was only performed in 30% of the 301 days.

As other intensive care unit (ICU) therapies, nutritional support has become more complex requiring tight supervision and monitoring. It has repeatedly been shown that despite awareness of guidelines and prescription of the recommended amounts of energy (25 kcal/kg), both overfeeding and underfeeding still remains a problemin ICU.

Research have shown that implementation of new guidelines has many barriers. Effective nurse physician collaboration may enhance the success of the compliance to the guidelines. Shared guidelines for nurses and physicians may lead to less complex decision-making of the patient care and enhanced quality of care.

